# Relationship between lacrimal gland changes and corneal parameters in patients with primary Sjögren’s syndrome and non-Sjögren’s syndrome-related dry eye

**DOI:** 10.3389/fmed.2026.1726563

**Published:** 2026-01-22

**Authors:** Wenyan Zhou, Chang Liu, Bo Yang, Jingyi Li, Haozhe Yu, Zhongqiang Yao, Xiaojun He, Shumin Wang, Yun Feng

**Affiliations:** 1 Department of Ophthalmology, Peking University First Hospital, Beijing, China; 2 Department of Ultrasound, Peking University Third Hospital, Beijing, China; 3 Department of Ophthalmology, Peking University Third Hospital, Beijing, China; 4 Department of Rheumatology and Immunology, Peking University Third Hospital, Beijing, China

**Keywords:** cornea nerve, dry eye disease, *in vivo* confocal microscopy, lacrimal gland ultrasonography, primary Sjögren’s syndrome

## Abstract

**Background:**

Primary Sjögren’s syndrome(pSS) is characterized as an autoimmune disorder mostly involving exocrine glands and pSS related dry eye (SSDE) contributes to a severe subtype of dry eye disease (DED). Emerging imaging tools for ocular surface evaluation such as lacrimal gland ultrasonography (LGUS) and *in vivo* confocal microscopy (IVCM) remain underutilized in diagnosing the SSDE. This study aims to investigate LGUS-IVCM correlations to map structural-functional relationships in SSDE patients.

**Methods:**

This prospective cross-sectional study enrolled 27 SSDE patients and 12 non-pSS related dry eye (NSSDE) controls, utilizing IVCM and LGUS to assess corneal nerve morphology, immune cell activity, and glandular structural parameters.

**Results:**

SSDE patients exhibited greater nerve tortuosity (*p* = 0.003), dendritic cell density (*p* < 0.001), and parenchymal echogenicity alterations (*p* = 0.013) versus NSSDE. For dry eye patients, subbasal nerve density inversely correlated with lacrimal gland area (*r* = −0.352, *p* < 0.05), logistic regression confirmed lacrimal gland area as an independent risk factor of nerve depletion. Meanwhile, ROC curve of dendritic cell density and activation strongly predicted SSDE with an AUC of 0.838 and 0.827.

**Conclusion:**

SSDE patients experience more corneal epithelial injury, with progressive lacrimal gland changes contributing to corneal nerve damage and inflammation. Multimodal ophthalmic imaging elucidates the interconnected lacrimal gland-corneal neuroimmune dysfunction in SSDE, offering potential diagnostic biomarkers and therapeutic targets for future exploration.

## 1 Introduction

Dry eye disease (DED) is a multifactorial disorder of the ocular surface associated with impaired ocular lubrication (1, 2). DED affects 5–30% of the population, with prevalence rising sharply with age. Approximately 10% of DED cases are related to underlying Sjögren’s syndrome (3–5). Primary Sjögren’s syndrome (pSS) is a chronic autoimmune disorder characterized by lymphocytic infiltration and damage to the exocrine glands, such as the salivary and lacrimal glands (LG) (6). The majority of pSS patients develop aqueous-deficient DED as a result of lacrimal gland dysfunction, manifesting as persistent ocular irritation, visual disturbance, and, in severe cases, corneal ulceration (1, 7). Tear film instability significantly affects the quality of life and serves as an important diagnostic marker for pSS, emphasizing the need to understand its underlying mechanisms (6, 8). DED can also develop independently of pSS. Based on this distinction, DED can be classified as pSS-related dry eye (SSDE) or non-pSS-related dry eye (NSSDE) (7).

Recent advancements in lacrimal gland ultrasonography (LGUS) have revolutionized the non-invasive assessment of gland morphology in pSS (9). High-resolution ultrasound enables precise quantification of parenchymal heterogeneity through grayscale analysis, whereas Doppler modalities effectively evaluate vascular patterns. Emerging evidence suggests that LGUS-derived parameters, including glandular volume reduction and hypoechoic foci, exhibit strong correlations with Schirmer’s test results and ocular surface disease index (OSDI) scores in SSDE patients (10, 11).

Concurrently, *in vivo* confocal microscopy (IVCM) offers high-resolution, real-time visualization of ocular surface structures, aiding the detection of disease-related changes (12, 13). IVCM has uncovered distinctive microstructural alterations in DED, including corneal nerve changes and ocular surface inflammation (14). Significantly reduced nerve fiber density and increased subbasal nerve plexus tortuosity have been observed in SSDE patients compared to healthy controls (15, 16).

However, current research has mainly focused on ocular surface changes in SSDE and NSSDE patients, overlooking the LG as the initial injury site in pSS (17). The potential interplay between macroscopic lacrimal gland structural changes and microscopic ocular surface damage remains unexplored. Integrating these imaging modalities could help map disease progression from glandular injury to clinical symptoms, uncover underlying mechanisms, and improve therapeutic monitoring in pSS-associated DED. Thus, this study aims to integrate LGUS and IVCM to quantify the association between lacrimal gland structural indices and corneal neuroimmune metrics.

## 2 Methods

### 2.1 Study design

This is a cross-sectional study of patients recruited from November 2023 to August 2024 at the Peking University Third Hospital. The study was reviewed and approved by the Scientific Research Ethics Committee of Peking University Third Hospital (IRB00006761-M2020456) before the study began. All patients in the study provided informed consent and complied with the tenets of the Declaration of Helsinki on human research.

### 2.2 Subjects

We recruited a total of 39 patients, comprising 27 with SSDE and 12 with NSSDE. The diagnostic criteria for dry eye and pSS were based on the definitions provided by the TFOS DEWS II and the 2016 American College of Rheumatology/European League against Rheumatism Classification Criteria for Primary Sjögren’s Syndrome (ACR/EULAR) (18, 19). All enrolled pSS patients were verified by the rheumatology and immunology departments. Subsequently, they were screened for dry eye and underwent IVCM and LGUS conducted by the ophthalmology and ultrasound departments. The inclusion criteria for pSS patients are as follows: (1) diagnosis of both pSS and DED, (2) no other systemic complications (such as purpura and peripheral neuropathy) or eye diseases (such as glaucoma), (3) age between 18 and 70 years, and (4) ability to cooperate with all ophthalmic examinations and clinical tests. Patients with a history of head and neck radiation therapy, active hepatitis C infection (positive PCR results), acquired immunodeficiency syndrome, sarcoidosis, amyloidosis, graft-versus-host disease, or IgG4-related disease were excluded. To eliminate potential confounding effects on the ocular surface, the enrolled patients did not receive any treatment with anti-inflammatory or lubricant medications.

### 2.3 System and ocular symptom assessment

First, we conducted serum antibodies tests (anti-SSA and anti-SSB) in the target patients and performed labial gland biopsies. Meanwhile, we assessed each patient’s EULAR Sjogren’s Syndrome Disease Activity Index (ESSDAI) and EULAR Sjogren’s Syndrome Patient Reported Index (ESSPRI) (20, 21). All serum analytes were detected using chemiluminescence immunoassays (Siemens AG, Germany) at Peking University Third Hospital. All participants completed the Ocular Surface Disease Index (OSDI), which consists of 12 questions and has a scoring range of 0–100 points. All participants were instilled with minimal volume fluorescein into the inferior temporal tear meniscus using fluorescein sodium ophthalmic test paper (Liaoning Meizilin Pharmaceutical Co., Ltd., China) for the evaluation of tear meniscus height (TMH), fluorescein tear break-up time (FBUT), and ocular staining score (OSS) during slit lamp examination. A Schirmer test strip (Liaoning Meizilin Pharmaceutical Co., Ltd., China) was placed on the outer one-third of the temporal lower conjunctival fornix for 5 min for each patient to perform the Schirmer I test after all other slit lamp examinations had been completed for at least 15 min. Corneal sensitivity was assessed by retracting the head while using a 6 cm-long soft, needle-like pencil to prompt a blink. If the patients did not blink, the needle was gradually shortened and tested until the first prompt blink occurred (22).

### 2.4 Ultrasound examinations

Lacrimal gland ultrasonography (LGUS) was performed on all patients by a single ultrasound physician blinded to clinical data. This physician had over 8 years of experience in superficial organ ultrasound examinations. A Samsung RS9 system (Samsung Medison Co., Ltd., Seoul, Korea) equipped with a 4–18 MHz multifrequency linear probe was used.

LGUS examinations were performed with the patients in a supine position, with their eyelids gently closed. The lacrimal region at the superolateral orbital margin was scanned obliquely using a plane nearly parallel to the anterior orbital contour, with part of the ocular globe serving as an acoustic window. Once the lacrimal gland was identified, the scanning plane was adjusted to the optimal orientation to maximize gland elongation and visualization. The long and short diameters of the gland were measured, and the contour was traced to calculate the area. Echogenicity of the gland parenchyma was assessed using a four-grade semi-quantitative scoring system, referencing the OMERACT-established evaluation method for salivary glands (23).

Grade 0 refers to a normal gland with uniform parenchymal echogenicity; Grade 1 indicates minimal changes, characterized by mild inhomogeneity without focal hypoechoic areas; Grade 2 represents moderate changes, manifested as moderate inhomogeneity with scattered focal hypoechoic areas; and Grade 3 signifies severe changes, demonstrated by diffuse inhomogeneity where hypoechoic areas occupy the entire gland, replacing normal parenchyma (Supplementary Figure S1). A diffuse fatty or fibrous gland can be qualitatively assessed, with the fatty gland corresponding to Grade 1 and the fibrous gland corresponding to Grade 3.

### 2.5 IVCM image examinations and data acquisition

IVCM was used to observe the microstructure of the cornea. All participants underwent imaging with a digital corneal confocal laser-scanning microscope of the central cornea using Heidelberg Retina Tomograph 3 with the sequence mode (HRT II RCM Heidelberg Engineering Inc., Heidelberg, Germany, Rostock Cornea Module). After topical anesthesia with 0.4% oxybuprocaine hydrochloride (Oftan Obucain, Santen Oy, Tampere, Finland), a drop of carbomer (Viscotears, CIBA Vision Europe Ltd., Southampton, UK) was used as a lubricant gel on the ocular surface and the disposable sterile polymethylmethacrylate cap (Tomo-Cap; Heidelberg Engineering GmbH, Dossenheim, Germany) to decrease applanation of the cornea and reduce compression artifacts. Two-dimensional pictures captured using the IVCM had a definition of 384 × 384 pixels over an area of 400 × 400 μm, with a lateral spatial resolution of 0.5 μm and a depth resolution of 1–2 μm. By adjusting the position of the laser scanning camera with the corneal contact cap, the target mapped on the cornea corresponded to the central and peripheral cornea.

**Figure 1:**
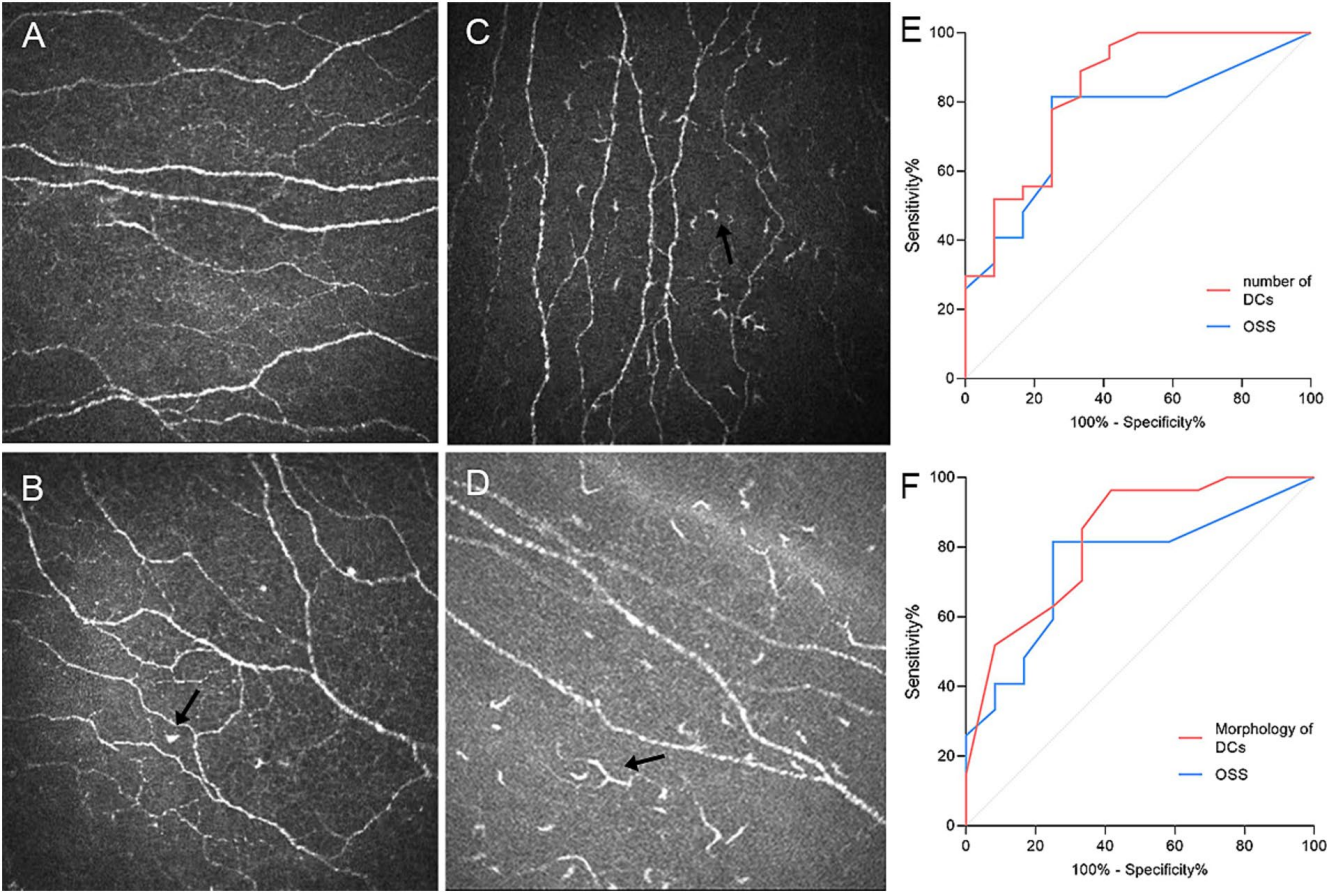
The DC size score and the ROC curve of DC parameters. Panels **A–D** show the DC size score based on a 3-point grading scale—**A**, Grade 0 (no dendritic cells); **B**, Grade 1 (globular cell: hyperreflective cell body without dendritic processes); **C**, Grade 2 (small cell body with two or fewer processes); **D**, Grade 3 (large cell body with more than two processes); and panels **E–F** show the ROC curve of dendritic parameters and OSS between SSDE and NSSDE groups. DC, dendritic cells/mm^2^.

An experienced ophthalmologist captured approximately 200–300 digital images within 5 min for each eye from the corneal epithelium to the endothelium. Among all the images, three right eye images of 40–70 μm with optimal quality without overlapping were selected by an experienced blinded observer for corneal subbasal nerve analysis. A Java-based image processing software (ImageJ, National Institutes of Health, Bethesda, MD) was used to collect morphological data of the corneal nerves, and dendritic cells were analyzed by the following parameters: (1) Density of nerves (mm/mm^2^): defined as the total length of the nerve fibers per square millimeter. (2) Reflectivity of nerves: defined as the average intensity of nerves. (3) Nerve tortuosity (grade 0–4): classified in four grades according to the previously reported scale (24) (Supplementary Figure S2). (4) Density of dendritic cells (/mm^2^): defined as the total number of dendritic cells per square millimeter. (5) Morphology of dendritic cells (grade 0–3): classified in three grades according to the activation of dendritic cells in the previously reported scale (25) (Figures 1A–D).

### 2.6 Statistical analysis

The statistical analysis was conducted using GraphPad Prism 9 and SPSS Version 23.0 software. Baseline data of patients, including the ocular surface, lacrimal gland ultrasound, and IVCM parameters, were derived from the right eye of each patient. Data distribution was assessed using the Shapiro–Wilk test. Continuous variables are expressed as mean ± SD for normally distributed data or as median (interquartile range) for non-normally distributed data. Categorical variables are presented as *n* (%). Student’s *t*-test or Mann–Whitney U test was used for group comparisons. Categorical variables were compared using Pearson’s chi-squared test. The Spearman correlation test was used to determine the relationships between the corneal subbasal nerve and dendritic cell grading and patients’ subjective symptoms. We used a generalized binary logistic regression model to estimate the association between the nerve density and other ocular data, with odds ratios (OR) and 95% confidence intervals (CI) for a higher bother score. Before launching the model, the Omnibus test and Hosmer–Lemeshow test were performed. The diagnostic performance of dendritic cells in distinguishing between the SSDE and NSSDE groups was evaluated using a receiver operating characteristic (ROC) curve. The ROC curve analysis included the calculation of the area under the curve (AUC) with 95% confidence intervals (CIs) to assess diagnostic accuracy. A *p*-value <0.05 was considered statistically significant.

## 3 Results

This study comprised 27 primary Sjögren’s-related dry eye (SSDE) patients and 12 non-Sjögren’s dry eye (NSSDE) controls (Table 1). All SSDE patients were confirmed through labial gland biopsy and rheumatologic evaluation, with 88.9% of patients having positive SSA antibodies. Although the groups were comparable in ages (49.41 ± 14.25 vs. 41.17 ± 15.95 years, *p* = 0.097) and similar overall dry eye severity, SSDE patients exhibited lower tear break-up time, reduced tear production, and diminished corneal sensitivity. Notably, ocular surface staining (OSS) scores were significantly higher in SSDE, reflecting more severe corneal epithelial damage. *In vivo* confocal microscopy (IVCM) revealed comparable subbasal nerve fiber density and reflectivity between groups. However, SSDE patients showed markedly increased nerve tortuosity and higher numbers and activation levels of dendritic cells. Lacrimal gland ultrasonography (LGUS) analysis identified significant differences in parenchymal echogenicity, while the largest transverse lacrimal gland sectional area did not differ significantly between the groups.

**Table 1:** Baseline parameters of the two groups.

Variable		pSS group (*n* = 27)	Non-pSS group (*n* = 12)	*p*
Basic information				
Age		49.41 ± 14.25	41.17 ± 15.95	0.097
Gender, female, %		27 (100)	11 (91.6)	0.134
IVCM				
Nerve fiber density, mm/mm^2^		10.88 ± 3.80	12.85 ± 3.14	0.073
Nerve reflectivity		130.46 (122.73, 141.92)	133.05 (130.95, 143.43)	0.181
Nerve tortuosity score		2.01 ± 0.68	1.53 ± 0.48	0.037*
Number of dendritic cells/mm^2^		58.33 (22.92, 112.50)	3.13 (0.52, 39.58)	0.001**
Dendritic cell morphology score		1.95 ± 0.67	0.94 ± 0.85	0.001**
Dry eye parameters				
BUT, s		2.15 (1.00, 3.25)	2.00 (1.25, 4.50)	0.284
Schirmer test, mm/5 min		5 (2, 10)	13 (1, 17)	0.350
TMH, mm		0.10 (0.05, 0.20)	0.10 (0.05, 0.18)	0.913
OSDI		22.92 (12.50, 37.50)	16.67 (12.50, 45.32)	0.604
OSS		4 (2, 11)	1 (0, 3.25)	0.012*
Corneal sensitivity		5.80 ± 0.62	6.00 ± 0	0.165
LGUS				
LG length, mm		7.40 (6.00, 8.70)	7.35 (5.78, 9.68)	0.927
LG width, mm		3.30 (2.80, 3.80)	3.20 (2.45, 3.85)	0.512
LG area, mm^2^		16.90 (11.21, 24.75)	16.43 (12.48, 23.72)	0.939
LG Grayscale score	0	5 (18.5)	3 (25)	0.033*
	1	6 (22.2)	7 (58.3)	
	2	9 (33.3)	2 (16.7)	
	3	7 (25.9)	–	
Diagnosis of pSS				
Anti-Ro, SSA, %		24 (88.9)	0 (0)	
Anti-La, SSB, %		11 (40.7)	0 (0)	
ANA positive, %		20 (74.1)	0 (0)	
Labial biopsy score	3	4 (14.8)	–	
	4	23 (85.2)		
ESSDAI		3.89 (2.13)	–	
ESSPRI		10.83 (6.68)	–	

Continuous variables are presented as mean (SD) when normally distributed and as median (interquartile range) when non-normally distributed. Categorical variables are presented as *n* (%). *p < 0.05, **p < 0.01.

**Figure 2:**
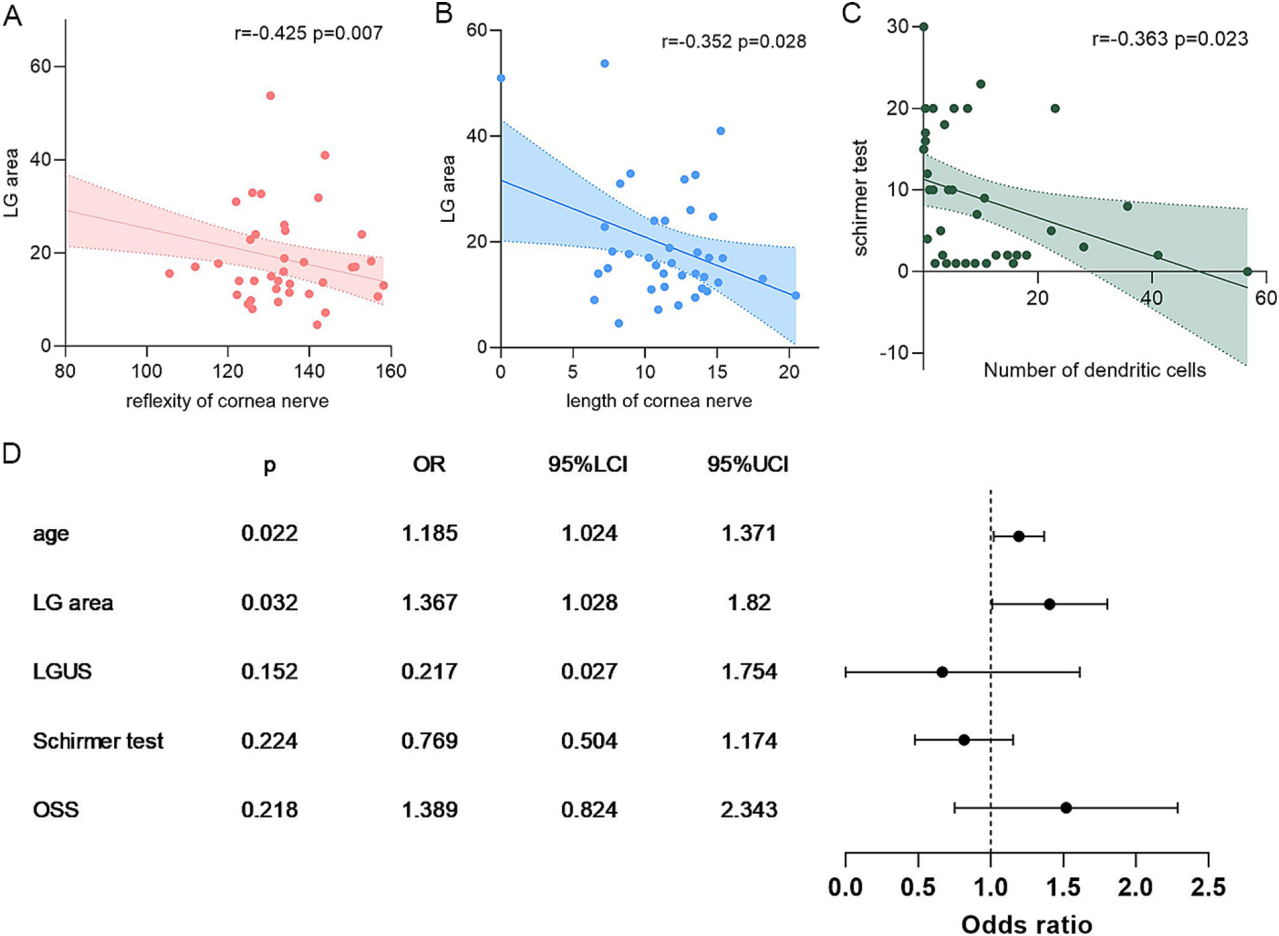
Correlation analysis of IVCM parameters, LGUS, and dry eye markers. Panels **A–B** show the correlation between subbasal nerve and LGUS parameters; **C**, the correlation between the number of DCs and the Schirmer test; and **D**, the binary logistic regression of subbasal nerve density. DC, dendritic cells/mm^2^; LGUS, lacrimal gland ultrasound; OSS, ocular staining score.

Correlation analyses identified significant associations between IVCM parameters and both lacrimal gland structure and clinical dry eye parameters (Table 2). Subbasal nerve density and reflectivity demonstrated strong negative correlations with lacrimal gland area (*r* = −0.352 and −0.425, *p* < 0.05, Figures 2A,B). Both subbasal nerve density and reflectivity correlated with corneal sensitivity (*r* = 0.565 and 0.736, *p* < 0.05). Reduced nerve length showed a trend toward association with higher OSS scores (*r* = −0.297, *p* = 0.066). Dendritic cell density and activation are inversely correlated with Schirmer test scores (*r* = −0.363 and −0.305, *p* = 0.023 and 0.051, Figure 2C). No meaningful correlations were detected between IVCM parameters and lacrimal gland echogenicity (grayscale score).

**Table 2:** Correlation between IVCM parameters and ocular evaluations.

Variable	Lacrimal gland area		Grayscale score		Schirmer test		OSS		TMH		Corneal sensitivity	
	*r*	*p*	*r*	*p*	*r*	*p*	*r*	*p*	*r*	*p*	*r*	*p*
Nerve fiber density (mm/mm^2^)	−0.352	0.028*	−0.012	0.944	0.244	0.144	−0.297	0.066	0.042	0.800	0.565	0.000**
Nerve reflectivity	−0.425	0.007**	−0.221	0.176	0.076	0.125	0.260	0.120	−0.168	0.307	0.736	0.000**
Nerve tortuosity score	0.241	0.145	0.032	0.851	−0.049	0.771	0.243	0.141	−0.013	0.936	−0.032	0.850
Number of dendritic cells (/mm^2^)	−0.154	0.349	0.154	0.351	−0.363	0.023*	0.272	0.094	−0.261	0.108	0.070	0.671
Dendritic cell morphology score	−0.228	0.162	0.083	0.615	−0.305	0.051	0.118	0.476	−0.169	0.305	0.167	0.310

*p < 0.05, **p < 0.01.

We further make a stratification of subbasal nerve density into two groups (<10 mm/mm^2^ vs. ≥10 mm/mm^2^, Figure 2D); multivariate binary logistic regression identified lacrimal gland area as an independent risk factor of nerve depletion (OR = 1.367, 95%CI: 1.028–1.820) (Univariate regression can be found in Supplementary Table S1). The regression model demonstrated statistical significance (Omnibus test *χ*^2^ = 27.01, *p* = 0.000) and demonstrated excellent goodness-of-fit (Hosmer–Lemeshow test *χ*^2^ = 1.434, *p* = 0.994). These findings suggest that larger lacrimal gland dimensions correlate with better-preserved corneal innervation in dry eye patients. Besides, receiver operating characteristic (ROC) curve analysis revealed that IVCM-measured dendritic cell density and activation demonstrated superior diagnostic performance for SSDE compared to ocular surface staining (OSS), with significantly higher area under the curve (AUC) values (0.838 vs. 0.753, *p* = 0.001, and 0.827 vs. 0.753, *p* = 0.013, respectively, Figures 1E,F).

## 4 Discussion

The demographic and clinical parameters between the SSDE and NSSDE groups align with previous reports (26). Although their Ocular Surface Disease Index (OSDI) scores were similar, SSDE patients exhibited significantly lower Schirmer test values, decreased tear film breakup time, reduced corneal sensitivity, and higher ocular surface staining (OSS) scores (27). This paradox suggests aqueous-deficient DED in pSS involves more severe inflammatory responses and neurosensory abnormalities beyond pure hyposecretion (28).

Our IVCM data between the two groups confirm two well-recognized phenomena in SSDE: (a) subbasal nerve fiber density reduction and distortion increase; (b) elevated dendritic cell density and activation. These align with Y. Matsumoto’s conclusion (29). The modest correlation with OSS scores, which reflect epithelial damage severity (*r* = −0.297, *p* = 0.066), and subbasal nerve fiber density may both result from epithelial injury. Corneal nerves, originating from the ophthalmic branch of the trigeminal nerve and terminating as delicate endings in the epithelium, provide innervation to each corneal epithelial and stromal cell (29). Consistently, decreased corneal sensitivity in SSDE has been demonstrated previously (30). Our results support a positive association between subbasal nerve fiber density and corneal sensitivity. However, nerve reflectivity in pSS patients contradicts previous reports. An observational study reported no significant changes in nerve reflectivity in SS patients was found (31). A statistically significant increase in tortuosity and reflectivity in patients with Sjogren’s syndrome compared to the control group was found (32). These differences may indicate either neurodegeneration or compensatory metabolic activation in response to epithelial abnormalities. The positive correlation between nerve reflectivity and corneal sensitivity warrants further investigation to elucidate the underlying mechanisms.

Ultrasound offers important clinical advantages as a non-invasive, affordable, and radiation-free imaging tool with high repeatability. Although limited experience with the scoring system in LGUS was reported, features like glandular parenchyma visibility, glandular size, and glandular parenchyma homogeneity demonstrated high consistency when assessed in images (33). We used high-frequency ultrasound to image the patients’ lacrimal glands. To our knowledge, this is the first study to explore the correlations between LGUS and IVCM metrics. We found that the lacrimal gland area has a negative relationship with subbasal nerve density, while the LG area may serve as a risk factor of nerve depletion, independent of age. Although research on gland size alterations in Sjögren’s syndrome remains limited, previous studies have reported significant parotidomegaly (34) and an enlarged lacrimal gland area detected via ultrasound (35) compared to non-Sjögren’s subjects. We propose that this gland enlargement may not represent an adaptation to dry eye but rather a pathological change, as a significant association between clinical parotidomegaly and a larger echographic surface has been reported (34). Previous research has revealed significantly reduced aquaporin 4 (AQP4) mRNA expression in the acinar and ductal cells of rabbits with autoimmune dacryoadenitis compared to healthy controls (36). AQP4 is a water channel protein that plays a critical role in fluid secretion and osmoregulation across cell membranes and is expressed in secretory epithelia (e.g., salivary and lacrimal glands). With functional loss, the mass of the gland turned into fatty tissue, with the development of dry eye (37), as well as the nerve length mentioned above. Besides, researchers have reported that corneal nerve loss in Sjögren’s dry eye has a relationship with reduced secretion of nerve-derived growth factors (29). It is also confirmed that members of the neurotrophin family of growth factors and their receptors exist in the rat lacrimal gland, which suggests a role for neurotrophins and their receptors in the lacrimal gland (38, 39). Residual acini may secrete fewer neurotrophic factors, which leads to corneal neuropathy. Besides, tear production and secretion from the lacrimal gland are regulated by a complex network of signaling pathways, including the MAPK/ERK and P38/JNK pathways (40). Oxidative stress activation, production of inflammatory mediators, and elevated levels of pro-inflammatory cytokines through the signaling pathways cause damage to the tear-producing glands and cornea simultaneously (28, 41, 42). However, we do not find any correlation between the LG grayscale score and corneal nerve; this may indicate that macroscopic structural changes in the lacrimal gland do not temporally coincide with neurodegenerative changes in the corneal subbasal nerve plexus.

Another marker that demonstrates corneal inflammation is dendritic cell density and activation status by IVCM. We found significantly elevated dendritic cell density and activation scores in SSDE patients compared to NSSDE controls, consistent with a previous study (17, 29). Moreover, dendritic cell density and activation strongly correlate with tear production, suggesting that decreased tear secretion and chronic inflammatory activation coexist during dry eye pathogenesis. Although reports on this relationship in pSS patients are limited, patients with epidemic keratoconjunctivitis associated with dry eye and contact lens wearers with dry eye also exhibit similar patterns (43, 44). While dendritic cell parameters demonstrated promising diagnostic value, with area under the curve (AUC) values of 0.838 and 0.827 in receiver operating characteristic (ROC) analyses, their clinical utility remains limited compared to ocular surface staining (OSS) because of technical complexity, operational demands, and higher costs. These limitations highlight the necessity of reserving IVCM for severe dry eye cases that require screening of Sjögren’s syndrome, particularly when autoimmune etiology is suspected. Future research should prioritize developing accessible biomarkers, such as rapid tear fluid assays quantifying inflammatory cytokines or dendritic cell-derived exosomes, to circumvent current imaging constraints while maintaining diagnostic accuracy (45).

However, this study has several limitations. The study’s limited cohort size reflects the relatively low prevalence of primary Sjögren’s syndrome in populations with dry eye. We used conventional clinical tests for dry eye assessment rather than non-invasive tear metrics. Although this choice is unlikely to have influenced our results, it may have reduced patient comfort. While our preliminary findings are encouraging, future trials with more participants are necessary to validate these findings and clarify the temporal relationships among lacrimal gland pathology, ocular surface inflammation, and neurodegeneration.

## 5 Conclusion

In summary, we observed a significantly elevated corneal staining score, increased density of dendritic cells in the cornea, and altered lacrimal gland echogenicity in SSDE patients. Meanwhile, we demonstrated the intricate relationship between lacrimal gland structural changes and ocular surface neuroimmune alterations. Our multimodal imaging approach highlights the interconnected lacrimal gland-corneal neuroimmune dysfunction, providing novel diagnostic biomarkers and potential therapeutic targets for SSDE patients.

## Data Availability

The raw data supporting the conclusions of this article will be made available by the authors, without undue reservation.

## Author’s note

The article has been preprinted on Research Square, after which we adjusted the abstract, and the language was polished according to the Author Guidelines of *Frontiers in Medicine*. doi: 10.21203/rs.3.rs-7147882/v1.

## Supplementary material

The Supplementary material for this article is available online at: https://www.frontiersin.org/articles/10.3389/fmed.2026.1726563/full#supplementary-material
