# Supplementary material for: Relationship between lacrimal gland changes and corneal parameters in patients with primary Sjögren’s syndrome and non-Sjögren’s syndrome-related dry eye
Source: Front Med (Lausanne). 2026 Jan 22;13:1726563. doi: 10.3389/fmed.2026.1726563 (PMC12872520; doi:10.3389/fmed.2026.1726563)
Supplement: Supplementary file 1 [file Data_Sheet_1.docx]

Supplementary Material

# Supplementary Figures and Tables

## Supplementary Table

**Table S1** Univariate and Multivariate logistic regression of Nerve length

| Variable | Univariate | | | | | Multivariate | | | | |
| --- | --- | --- | --- | --- | --- | --- | --- | --- | --- | --- |
|  | β | S.E. | Z | P | OR(95%CI) | β | S.E. | Z | P | OR(95%CI) |
| Age | 0.09 | 0.03 | 8.02 | 0.005* | 1.094(1.028, 1.165) | 0.17 | 0.07 | 5.21 | 0.022* | 1.185(1.024, 1.371) |
| LG area | 0.09 | 0.04 | 4.87 | 0.027* | 1.090(1.010, 1.176) | 0.31 | 0.15 | 4.61 | 0.032* | 1.367(1.028, 1.82) |
| LGUS | 0.03 | 0.35 | 0.01 | 0.942 | 1.026(0.514, 2.049) | -1.53 | 1.07 | 2.05 | 0.152 | 0.217(0.027, 1.754) |
| Schirmer test | -0.13 | 0.07 | 4.06 | 0.044* | 0.876(0.771, 0.996) | -0.26 | 0.22 | 1.48 | 0.224 | 0.769(0.504, 1.174) |
| OSS | 0.13 | 0.08 | 2.41 | 0.120 | 1.133(0.968, 1.328) | 0.33 | 0.27 | 1.52 | 0.218 | 1.389(0.824, 2.343) |

* p < 0.05

## Supplementary Figures


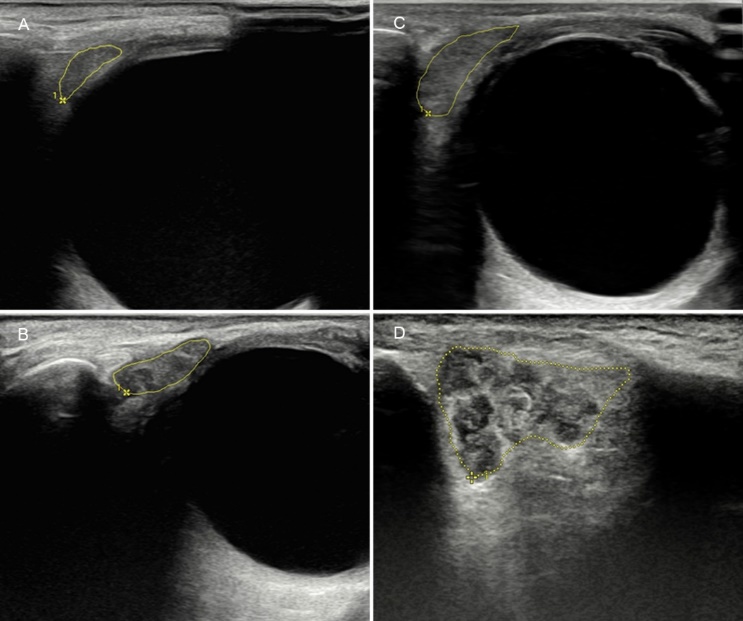


**Supplementary Figure 1.** Representative LGUS images illustrating the semi-quantitative greyscale scoring: (A) grade 0, normal parenchyma; (B) grade 1, minimal change; (C) grade 2, moderate change; (D) grade 3, severe change.


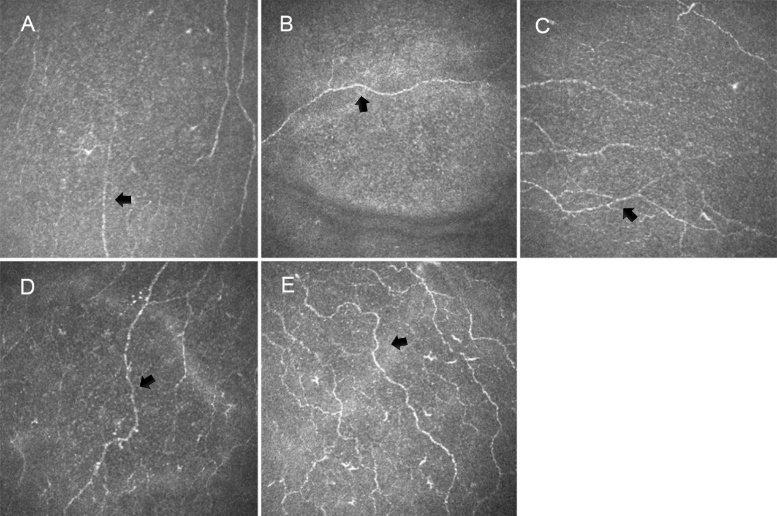


**Supplementary Figure 2.** The nerve tortuosity score based on a 5-point grading scale A, Grade 0. Almost straight. B: Grade 1. Slightly tortuous. C: Grade 2. Moderately tortuous with small amplitude of changes in the direction. D: Grade 3. Quite tortuous with severe amplitude of the changes in the fiber direction. E: Grade 4. Very tortuous with abrupt and frequent changes in the nerve fiber direction.
